# Molar Pregnancy Presents as Tubal Ectopic Pregnancy

**Published:** 2011-02-20

**Authors:** Fatemeh Davari Tanha, Elham ShirAli, Haleh Rahmanpour, Fediey Haghollahi

**Affiliations:** 1 Department of Obstetrics and Gynecology, Tehran University of Medical Sciences, Tehran, Iran; 2 Department of Obstetrics and Gynecology, Zanjan University of Medical Sciences, Zahadan, Iran; 3 Department of Obstetric and Gynecology, Vali Asr Reproductive Health Center, Tehran University of Medical Sciences, Tehran, Iran

**Keywords:** Hydatidiform Mole, Ectopic Pregnancy, Choriocarcinoma

## Abstract

Hydatidiform moles are abnormal gestations characterized by the presence of hydropic changes affecting some or all of the placental villi. Hydatidiform moles arise as a result of the fertilization of an abnormal ovum. In this report, the patient was a 29 year old Asian woman who had induction of ovulation with letrozol. Since the majority of molar gestations arise within the uterine cavity thus the occurrence of a hydatidiform mole within ectopic gestational tissue is rare. It is important to differentiate a hydatidiform mole from a conventional ectopic pregnancy, particularly in infertile women who have a history of ovulation induction.

## Introduction

Hydatidiform moles are abnormal gestation characterized by the presence of hydropic changes affecting some or all of the placental villi. Hydatidiform moles arise as a result of fertilization of an abnormal ovum of which the majority originate within the uterine cavity. The occurrence of a hydatidiform mole within ectopic gestational tissue is rare (1).

## Case report

The patient was a 29 year old Asian woman from Iran who was referred to the Women’s Hospital in February 2007 due to a missed period (gestational age: eight weeks) and elevated human chorionic gonadotropin β (β-hCG) titer (15000 units/ml). Her gynecologic history was unremarkable except for primary infertility of one year’s duration due to polycyctic ovary syndrome. The patient’s pregnancy occurred with the use of letrozol. She was having vaginal bleeding since six days prior to admission with the passage of a clot and associated pelvic pain. Her past medical and surgical histories were unremarkable. She was a nonsmoker and had no allergies.

**Fig 1:**
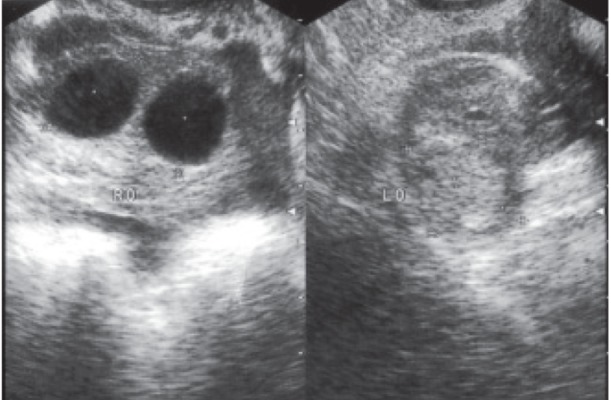
Left adnexal mass and right ovarian simple cyst.

**Fig 2:**
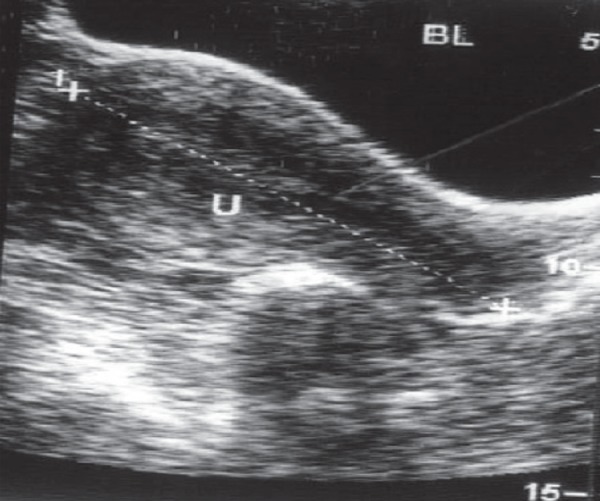
The uterine cavity with no gestational sac

The patient underwent a physical examination; blood pressure was 90/60, pulse 110 and a temperature of 37°C. The chest was clear and the electrocardiography (ECG) was normal. The patient had left lower quadrant (LLQ) tenderness by abdominal palpation . There was brown blood in the vagina. Internal examination revealed a retroverted uterus with left adnexal masse. Tenderness in the left adnexa and cervical motion tenderness were present. The patient underwent ultrasonography. There was no gestational sac in the uterus; the endometrial thickness was 9 mm, a left adenexal mass that consisted of a suspicious echofree area gestational sac (GS) of 18×28 mm and free fluid in the cul-de-sac were noted (Figs 1, 2).

The patient underwent laparotomy. The fallopian tube was resected due to a rupture that extended to the subserosal surface. Pathologic report was left fallopian tube ectopic pregnancy with features of a partial hydatidiform mole.

Based on the pathologic report, a workup for hydatidiform mole was begun, followed by serum β-hCG titer.

## Discussion

The incidence of a partial or complete hydatidiform mole in pregnancies is 1 in 500-1000 (1). Theoretically, the same proportion of ectopic pregnancies should also be affected by molar change since the main etiologic factor preceding both partial and complete hydatidiform moles is an abnormal androgenetic chromosomal constitution of the conceptus that is present before implantation regardless of the site (2). Tubal ectopic hydatidiform moles are rare occurrences and only 132 cases have been reported in the literature (2). The mean gestational age at admission was eight weeks (3). To the best of our knowledge, this is the first time a diagnosis of hydatidiform mole during early tubal pregnancy was made after the induction of ovulation with letrozol in an infertile woman.

All patients who present with a hydatidiform mole complain of abdominal pain; some also have vaginal bleeding. The condition can mimic the usual symptoms of ectopic pregnancy particularly when a hem peritoneum is present however it is actually an ectopic molar pregnancy (3).

The most cardinal diagnostic feature is the presence of a definite abnormal, nonpolar trophoblast proliferation that is circumferential in nature, usually presenting with a vacuolated phenotype and which may be associated with sheets of pleomorphic extravillus trophoblast fragments (4). Immunohistochemical markers such as P57KIP2, which has been recently described, can also be useful for diagnosing early moles even on the basis of minimal tissue since this protein is not expressed in the villus trophoblast or the stroma of complete hydatidiform moles (5).

Because trophoblastic tissue have an invasive nature when located in the early gestational sac, an ectopic pregnancy may be associated with apparent local invasion of surrounding tissues by the trophoblast (4).

The lesions of gestational trophoblastic tumor (GTT) misdiagnosed as an ectopic pregnancy can be seen in the fallopian tube, horn of the uterus, peritoneal cavity, greater omentum and recto - uterine pouch (2). Misdiagnosis leads to delay in therapy with resultant increased morbidity of GTT (6). However, none of the cases in one series developed persistent gestational trophoblastic disease, and hCG concentrations spontaneously returned to normal levels during surveillance in all cases that had a confirmed diagnosis of hydatidiform mole (4).

However most other previously described cases did not develop persistent gestational trophoblastic disease (GTD) clinically or require chemotherapy. Consequently, the risk for persistent GTD after an extra-uterine molar gestation is approximately 0.5% for partial and 15% for complete hydatidiform moles. The diagnosis of apparently primary tubal choriocarcinoma with no confirmed previous ectopic hydatidiform mole is now wellreported but poses no specific histopathologic diagnostic problems; the features are identical to choriocarcinoma at other sites (4). In many cases metastatic disease may be present at diagnosis, but it remains unclear in what proportion of cases the choriocarcinomal may have developed from a previous unrecognized tubal molar conceptus or whether some cases may represent seeding from a uterine primary conception (6).

Patients who have received methotrexate for ectopic pregnancy are managed nonsurgically because no tissue diagnosis is available. hCG monitoring to ensure return to normal levels is suggested.

## Conclusion

A tubal ectopic hydatidiform mole is a rare condition. The mean gestational age at admission is eight weeks. It is important that after induction of ovulation for infertility treatment, the clinician considers the possibility of a hydatidiform mole in the extra-uterine cavity of which special attention and treatment is needed, rather than simply treating an ectopic pregnancy. Additionally, in patients with histories of infertility and induction of ovulation, ectopic pregnancy is more common. It is possible that a rare presentation such as the hydatidiform mole which mimics an ectopic pregnancy is not rare.
